# The Prevalence of Violence Against Resident Doctors and Its Subsequent Effects on Patient Management in a Tertiary Care Hospital in Delhi, India

**DOI:** 10.7759/cureus.39116

**Published:** 2023-05-17

**Authors:** Aninda Debnath, Md. Alam, Mohit Goyal, Anita Khokhar, Shveta Lukhmana

**Affiliations:** 1 Community Medicine, Vardhman Mahavir Medical College and Safdarjung Hospital, Delhi, IND

**Keywords:** trauma, verbal abuse, physical abuse, doctors, workplace violence

## Abstract

Introduction

Workplace violence (WPV) is a significant problem for healthcare professionals across the world, regardless of whether they work in developed or developing countries. Studies have shown that in India, up to 75% of doctors have experienced some form of violence in the workplace. The purpose of the present study was to examine the extent of violence against doctors and its impact on patient management.

Methodology

This cross-sectional study was conducted in a tertiary care hospital in New Delhi in June 2022. A total of 326 resident doctors from six departments were selected using stratified random sampling. Data were collected using a semi-structured interview schedule and a pre-validated questionnaire. Statistical analysis was done using Stata 17, and ethical clearance was obtained from the Institute Ethical Committee.

Result

Workplace violence was prevalent among healthcare professionals, with 80.4% (95% confidence interval (CI): 75.6%-84.5%) experiencing verbal abuse and 21.7% (95% CI: 17.4%-84.5%) experiencing physical violence. Perceived delays in treatment and patient deaths were the most common causes of violence. Most participants were hesitant to report WPV due to time-consuming reporting processes and a lack of organisational support. WPV had a negative impact on doctors' mental and personal well-being, with 73.3% reporting its negative impact. WPV has led to a decrease in the provision of surgical and medical interventions.

Conclusion

The study findings suggest that a significant proportion of doctors in a tertiary care hospital in Delhi encounter some form of workplace violence. Despite the high incidence of WPV, reporting of these events remains low due to inadequate support and deficient reporting procedures within healthcare organisations. The negative impact of WPV is not limited to the physicians' psycho-social well-being but extends to their approach to patient care as well. Therefore, taking appropriate actions to prevent WPV is crucial for ensuring the safety and well-being of healthcare professionals and improving patient outcomes.

## Introduction

Workplace violence (WPV) is a pernicious issue encompassing a spectrum of violent behaviours and threats directed towards workers, occurring both on and off the job [1]. These behaviours can include verbal tirades, physical assault, and even homicide and can affect workers from all healthcare disciplines. In developing countries, WPV has had a particularly deleterious impact on healthcare systems, compromising their overall efficacy [2].

The issue of violence against doctors is not a regional anomaly but a global predicament [3]. In the United States, studies from as far back as the 1980s have indicated that more than half of emergency care workers have been threatened with a weapon [4]. Similarly, in the United Kingdom, nearly half of doctors have reported experiencing some form of aggression [5]. In Asian countries such as China, Israel, Pakistan, and Bangladesh, the prevalence of violence against medical professionals has been reported to be higher compared to Western countries [6]. According to the Indian Medical Association, up to 75% of doctors in India have encountered some form of violence in the workplace, which is comparable to rates observed in other countries in the region [7]. This highlights the pressing need for strategies to address WPV against healthcare workers, both in India and globally.

Violence is the second-leading cause of death in the workplace [8]. One of the far-reaching effects of workplace violence on healthcare is the escalation of healthcare costs [9]. Violence costs both the victim and the authorities. Factors that have so far been identified as being associated with WPV include working in remote healthcare areas, understaffing, the mental and emotional stress of patients and visitors, insufficient security, and a lack of preventative measures associated with WPV against healthcare professionals [10].

The Indian government has addressed the growing issue of violence against medical practitioners by introducing various state government acts and the Disease Ordinance Amendment Act 2020 [1]. Despite these measures, incidents of violence against doctors continue to increase, making it essential to understand the significance of this problem. Previous studies have primarily focused on estimating the prevalence of violence against doctors, but there is a lack of literature describing the impact of such violence on patient management. Moreover, most of the available studies were conducted before the COVID-19 pandemic, and it is unclear whether the trend of violence against doctors has changed since then. The current study aims to fill this research gap by assessing the prevalence of violence against doctors and its impact on patient management.

## Materials and methods

Study setting

We conducted this cross-sectional study in June 2022 in a tertiary care hospital in New Delhi.

Study population and sampling strategy

We included non-academic junior residents (JR), postgraduate residents (PG), and senior residents (SR) of the hospital with working experience of at least six months in the study. A sample size of 330 was calculated, taking the prevalence of workplace violence against doctors as 77%, a 95% confidence interval (CI), a 5% relative error, and a non-response rate of 10% [10]. We have used stratified random sampling in our study. To ensure a representative sample, we used stratified random sampling and conveniently selected six departments, including medicine, casualty, surgery, obstetrics and gynaecology, orthopaedics, and paediatrics. Among these departments, three were surgical (surgery, obstetrics and gynaecology, and orthopaedics), and the other three were non-surgical (medicine, paediatrics, and causality). We created a list of non-academic JR, PG, and SR from each department to form our sampling frame. We selected 55 participants from each department's sampling frame by simple random sampling using a computer-generated random number.

Study instrument

We used a pre-tested, semi-structured, and self-administered interview schedule with three sections to collect the data.

Section 1: This section included information on the socio-demographic attributes of the study participants, such as their educational background and work-related details.

Section 2: In the second part, we assessed workplace violence using a pre-validated questionnaire. The questionnaire was validated in India and is available for free use. The questionnaire comprised five domains to assess the burden of the problem, associated risk factors, and mitigation strategies to solve it [11].

Section 3: This part focused on the subsequent effect of violence on doctors on patient management. It was developed after a detailed review of studies.

Study process

All the participants were contacted by the authors, MA and MG, and interviewed after obtaining informed consent. If any participant could not be contacted after three visits, they were marked as non-respondents.

Working definitions

Workplace Violence (WPV)

Workplace violence was defined as any event where workers were subjected to abuse, threats, or assault in connection with their work, including while commuting to or from work, which poses an explicit or implicit threat to their safety, well-being, or health. Such incidents can take various forms, such as physical assault, homicide, verbal abuse, bullying or mobbing, sexual and racial harassment, and psychological stress [1].

Physical Violence

The use of physical force against another person or group results in physical, sexual, or psychological harm. It includes beating, kicking, slapping, stabbing, shooting, pushing, biting, and pinching, among others [1].

Verbal Abuse (VA)

Verbal abuse was characterised as the subjective experience of being subjected to professional or personal attacks, devaluation, or humiliation through spoken language. This type of abuse could take various forms, such as the use of derogatory remarks, abusive or offensive language, and profane or obscene comments [1].

Statistical analysis

Data were entered into Microsoft Excel and analysed using Stata Statistical Software, Release 17 (2022, Stata Corp. LP, College Station, Texas). The prevalence of workplace violence was expressed as a percentage and presented with corresponding 95% confidence intervals. The statistical significance of differences in proportions related to specific factors was determined using the Chi-square test, and in the case of an expected frequency of less than five, the Fischer exact test was employed. A p-value of less than 0.05 was considered statistically significant.

Ethical considerations

We have obtained ethical clearance from the Institute Ethics Committee. (Ref no. IEC/VMMC/SJH/Project/06-2022/CC-02.) Before participating in the study, each participant received a briefing on the study's objectives and was provided with an information sheet. All participants were required to provide informed written consent before taking part in the study.

## Results

Sociodemographic profile

The present study involved 326 participants who took part in the research. Of the total number of respondents, 198 (60.7%) were men, and the remaining 128 (39.3%) were women. The participants' mean age was 27.3 years, with a standard deviation of 4.6 years. Most participants were unmarried or single (n= 256, 78.5%), while only 70 (21.5%) were married. The median duration of employment at the institute was three years, with an interquartile range (IQR) of one to four years. The participants were mostly junior residents (n = 182; 56%), followed by senior residents (n = 143; 44%) (Table 1).

**Table 1:** Sociodemographic and professional characteristics of the study participants * Chi-square test WPV: workplace violence

Variables	WPV faced n = 262	WPV not faced n = 64	Total N =326	p-value
Gender				
Male	161(61.5%)	37(57.8%)	198(60.7%)	0.59
Female	101(38.5%)	27(42.2%)	128(39.3%)	
Age (years)				
Less than 30 years	223(85.1%)	52(81.2%)	275(84.4%)	0.44
30 years or more	39(14.9%)	12(18.8%)	51(15.6%)	
Marital status				
Married	55(20.9%)	15(23.4%)	70 (21.5%)	0.6
Unmarried	207(79.1%)	49(76.6%)	256 (78.5%)	
Highest qualification				
MBBS	144(55.2%)	38(59.4%)	182 (56%)	0.5
Postgraduate (MD/MS/DM/MCH/DNB)	117(44.8%)	26(40.6%)	143 (44%)	
Department				
Non-surgical department	147(56.1%)	20(31.3%)	167(51.2%)	0.01*
Surgical department	115(43.9%)	44(68.7%)	159(48.8%)	
Years of experience				
Less than 1 year	72(27.5%)	14(21.9%)	86(26.4%)	0.26
1 year – 5 Years	166(63.3%)	47(73.4%)	213(65.3%)	
5 years or more	24(9.2%)	3(4.7%)	27(8.3%)	

Prevalence of workplace violence

Out of the 326 participants, 262 reported that they had at least one incidence of workplace violence in their lifetime, resulting in the prevalence of WPV being 80.4% (95% CI: 75.6%-84.5%). The prevalence of workplace physical violence was 21.7% (n =71) (95% CI: 17.4%-26.6%). Those participants who had faced verbal abuse also faced physical violence in the workplace. The prevalence of verbal abuse was 80.4% (95% CI: 75.6%-84.5%).

The study found that the prevalence of harassment was higher among non-surgical departments than among surgical departments (p-value of 0.01) (Table 1). Among the departments analysed, verbal abuse was most common in the paediatrics department, where 96.2% of doctors reported experiencing it. In contrast, physical violence was most common in the casualty department, with 29.1% of doctors reporting experiencing it.

**Figure 1:**
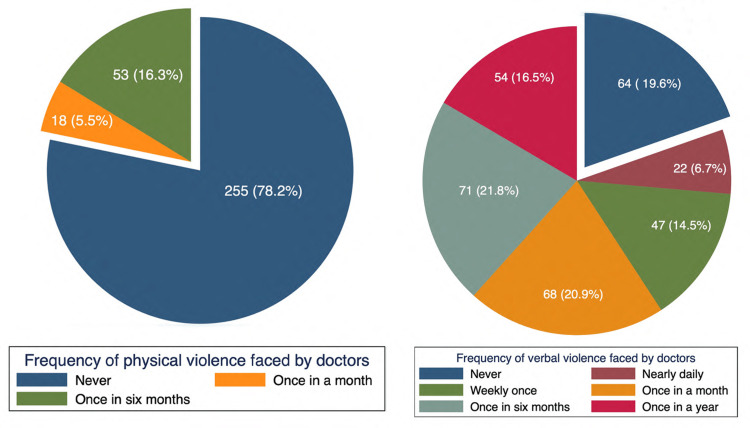
(A) Frequency of verbal violence faced by doctors; (B) Frequency of physical violence faced by the doctors Image created by the authors.

The study found that 22 (6.7%) participants reported experiencing verbal violence on a daily basis, and 14.5% reported experiencing it at least once a week. Additionally, 20.9%, 21.8%, and 16.5% of participants reported experiencing verbal violence at least once a month, once every six months, and once a year, respectively. Regarding physical violence, 18 (5.5%) participants reported experiencing it once a month, while the remaining 53 (16.5%) participants reported experiencing it once every six months or less (Figure 1).

The perceived causes of violence 

**Figure 2:**
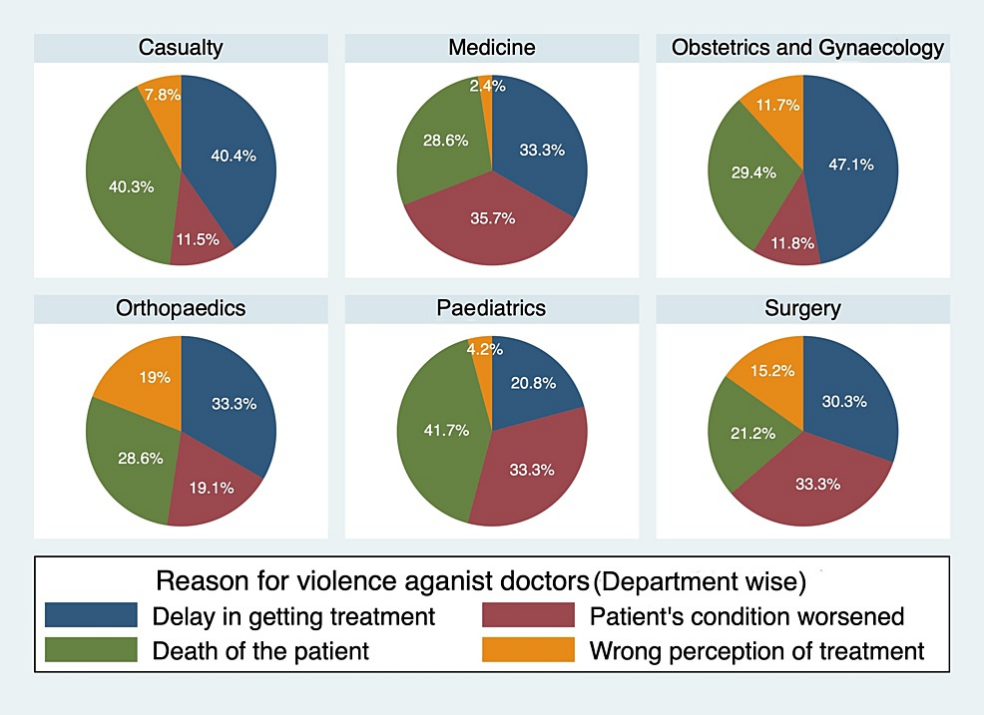
Reasons for violence against doctors, organised as per the department Image created by the authors.

The study revealed that the primary cause of workplace violence among healthcare professionals was either an actual or perceived delay in receiving medical treatment, which accounted for 33.8% of all reported incidents. The death of a patient was the second most commonly reported cause in 32.6% of cases. Other significant factors included non-improvement or deterioration of the patient's condition (23.9%) and incorrect perceptions about treatment (9.7%). Further analysis showed that the most common cause of violence in the casualty, obstetrics and gynaecology, and orthopaedics departments was the delay in getting treatment while worsening patients' conditions was the most common cause in the medicine and surgery departments. Additionally, the death of the patient was the most common cause of violence reported in the paediatrics department (Figure 2).

Reporting pattern of WPV among doctors 

In our study, we examined the reporting behaviour of participants towards WPV and identified the factors that hindered the reporting of such incidents. A total of 244 participants (74.8%) reported feeling comfortable reporting WPV events, while the remaining participants were either neutral or uncomfortable. The most common reason cited by the participants for not reporting WPV incidents was the perception that it was a time-consuming process (90.2%). Additionally, 89.9% of participants believed that no action would be taken if they reported WPV and 89.6% reported a lack of organisational support as a hindrance. Another factor that prevented participants from reporting WPV was the absence of reporting provisions (87.4%). Further, a significant proportion of participants (61.7% and 68.1%) felt ashamed of reporting and believed that reporting WPV incidents would affect their appraisal or promotion opportunities. Notably, the feeling of shame associated with reporting WPV was significantly associated with the violence faced (p-value <0.01) (Table 2).

**Table 2:** Association between WPV and factors involved in reporting WPV * Statistically significant, Chi-square test WPV: workplace violence

	Reason for reporting workplace violence		
Verbal abuse	Significant	Insignificant	p-value
	Felt ashamed of reporting		
Yes	152 (58%)	110 (42%)	0.01*
No	49 (76.6%)	15 (23.4%)	
	A belief that no action will be taken		
Yes	236 (90.1%)	26 (9.9%)	0.81
No	57 (89.1%)	7 (10.9%)	
	Lack of organizational support		
Yes	233 (88.9%)	29 (11.1%)	0.44
No	59 (92.2%)	5 (7.8%)	
	Lack of provision for reporting such incidences		
Yes	228 (87.1%)	34 (12.9%)	0.65
No	57 (89.1%)	7 (10.9%)	
	Time-consuming process		
Yes	234 (89.3%)	28 (10.7%)	0.28
No	60 (93.7%)	4 (6.3%)	
	Appraisal or promotion avenues will be affected		
Yes	175 (66.8%)	87 (33.2%)	0.30
No	47 (73.4%)	17 (26.6%)	

Workplace violence and its impact on the psycho-social lives of the participants

In the current study, it was found that a majority of participants, specifically 73.3%, reported that workplace violence (WPV) had a negative impact on their mental well-being. Additionally, 69.9% of participants reported that WPV had affected their personal well-being. Furthermore, social life and family were negatively affected by WPV, as reported by 59.2% and 53.4% of participants, respectively. These findings were statistically significant (p-value <0.01) and demonstrated a strong association between workplace violence and its impact on various aspects of doctors' lives. Participants who experienced workplace violence (WPV) exhibited a statistically significant inclination to contemplate changing their occupation (p < 0.01) and reported decreased motivation (p < 0.01) (Table 3-4).

**Table 3:** Association of the work-life impact of workplace violence with participants' experiences of workplace violence * Statistically significant, Chi-square test

Verbal abuse	Yes	No	p-value
	Thought of changing occupation		
Yes	95 (36.3%)	167 (63.7%)	0.01*
No	9 (14.1%)	55 (85.9%)	
	Felt like not working at all		
Yes	29 (11.1%)	233 (88.9%)	0.05
No	2 (3.1%)	62 (96.9%)	
	Thought of an alternate carrier		
Yes	92 (35.1%)	170 (64.9%)	0.12
No	16 (25%)	48 (75%)	
	Motivation decreased		
Yes	156 (59.5%)	106 (40.5%)	0.01*
No	8 (12.5%)	56 (87.5%)	

**Table 4:** Association of the psycho-social impact of workplace violence with participants' experiences of workplace violence * Statistically significant, Chi-square test

Verbal abuse	Not affected	Mildly affected	Moderately affected	Severely affected	p-value
	Affected personal well-being				<0.01*
Yes	51 (19.5%)	147 (56.1%)	58 (22.1%)	6 (2.3%)	
No	47 (73.4%)	16 (25%)	1 (1.6%)	0 (0%)	
	Affected family life				<0.01*
Yes	102 (38.9%)	115 (43.9%)	40 (15.3%)	5 (1.9%)	
No	50 (78.1%)	13 (20.3%)	1 (1.6%)	0 (0%)	
	Affected social life				<0.01*
Yes	82 (31.3%)	130 (49.6%)	42 (16%)	8 (3.1%)	
No	51 (79.7%)	12 (18.7%)	1 (1.6%)	0 (0%)	
	Affected mental well-being				<0.01*
Yes	40 (15.3%)	129 (49.2%)	77 (29.4%)	16 (6.1%)	
No	47 (73.4%)	16 (25%)	1 (1.6%)	0 (0%)	

The influence of WPV on subsequent patient management

The incidence of WPV against physicians has had a discernible influence on patient care and clinical decision-making. Notably, there has been a significant reduction in the administration of surgical and medical treatments, as well as the management of emergency cases, following instances of violence directed at doctors (p-value 0.04, <0.01). Conversely, the frequency of investigations suggested (p-value <0.01), consultation liaising with other specialties and referrals (p-value <0.01), consultation time (p-value <0.01), and preference for private hospitals over government hospitals (p-value <0.01) have increased in response to violence against doctors. Notably, the handling of non-complicated/non-emergency cases and prescribing patterns have not been significantly impacted by violence against doctors (Table 5).

**Table 5:** Impact of WPV on patient management and decision-making by the doctors who faced workplace violence * Statistically significant, Chi-square test

Verbal abuse	Increased	Decreased	p-value
	Prescribing drugs		
Yes	49 (18.7%)	213(81.3%)	0.76
No	13(20.3%)	51(79.7%)	
	Surgical or medical intervention		
Yes	82(31.3%)	180(68.7%)	0.04*
No	12(18.8%)	52(81.2%)	
	Suggesting investigations		
Yes	167(63.7%)	95(36.3%)	0.01*
No	27(42.2%)	37(57.8%)	
	Handling emergencies or critical cases		
Yes	123(46.9%)	139(53.1%)	0.01*
No	17(26.6%)	47(73.4%)	
	Handling non-complicated cases		
Yes	35(13.4%)	227(86.6%)	0.38
No	6(9.4%)	58(90.6%)	
	Handling and consultation liaising with other specialties		
Yes	163(62.2%)	99(37.8%)	0.01*
No	26(40.6%)	38(59.4%)	
	Consultation time		
Yes	146(55.7%)	116(44.3%)	0.01*
No	25(39.1%)	39(60.9%)	
	Preference of private over government hospitals		
Yes	161(61.5%)	101(38.5%)	0.01*
No	26(40.6%)	38(59.4%)	

## Discussion

Our study revealed that out of the 326 participants, 262 reported that they had at least one incident of workplace violence, resulting in the prevalence of WPV being 80.4% (95% confidence interval (CI): 75.6%-84.5%). The prevalence of workplace physical violence was 21.7% (n =71) (95% CI: 17.4%-26.6%). The prevalence of verbal abuse or violence was similar to workplace violence.

Our study revealed that 80.4% of resident doctors had experienced some form of WPV, which is considerably higher than the 35% reported by Kumari et al. in their study conducted in New Delhi in 2021 [11]. Similarly, studies conducted by Grover et al., Sharma et al., and Anand et al. also reported a lower prevalence of WPV among doctors compared to our study [12-14]. The differences in the prevalence rates of workplace violence across various studies may be explained by variations in the length of time that individuals were exposed to the phenomenon, the definition of workplace violence employed, and the geographic location of the study population. Notably, these studies used a variety of questionnaires, with Grover et al. using the World Health Organization's Workplace Violence in the Health Sector Questionnaire, while other studies used their own self-developed questionnaires. In our study, we used a validated questionnaire to assess workplace violence. However, the results of our study are in line with those from Kaur et al., Jain et al., who carried out pan-India surveys, and Singh et al. in Uttar Pradesh, who also reported comparable patterns of workplace violence against doctors [10,15,16].

The nature of workplace violence is a crucial aspect to consider when investigating the prevalence and impact of such incidents. In our study, verbal violence was the most common type of workplace violence reported by resident doctors, accounting for 80.4% of the incidents, while physical violence accounted for 21.7%. These findings are consistent with the results of a study by Ori et al. conducted among postgraduate students of a tertiary care hospital in Manipur, as well as studies by Singh et al. and Sharma et al., where verbal abuse was more frequent than physical violence [17-18]. However, our study reports a higher proportion of physical abuse compared to studies conducted by Vaishali et al. in Haryana and Sharma et al. in Gujarat [18,19]. Nevertheless, our findings align with the study conducted by Singh et al. [16]. The underlying reasons for the escalation of verbal abuse into physical violence may be attributed to the lack of safety measures on hospital premises.

In our study, the maximum incidence of verbal violence was reported by paediatric residents, while emergency department residents had the most episodes of physical violence. Previously done Indian studies unanimously show the emergency or casualty department faces the maximum episodes of violence, both verbal and physical [14,20,21].

The study conducted by Kumar et al. showed that extended waiting periods (73.5%), delayed medical provision (45.6%), violation of visiting hours, and patient dissatisfaction with care (41%), were the most prevalent factors that contributed to violent incidents [21]. Similarly, Kaur et al. identified non-improvement or worsening of the patient's condition, misperception of treatment (37.3%), patient's death (34.4%), and actual or perceived delay in treatment (28.5%) as the fundamental reasons for violence in healthcare facilities [10]. Likewise, our study established that perceived delay in treatment (33.8%), patient mortality (32.6%), patient health deterioration (23.9%), and erroneous perception of treatment (9.7%) were potential triggers for violence.

Our study revealed that 25.2% of participants were hesitant to report WPV incidents, citing a perception that the reporting process was time-consuming (90.2%), that no action would be taken if they reported WPV (89.9%), a lack of organisational support (89.6%), and the absence of reporting provisions (87.4%) as hindrances. In comparison, Kaur et al. and Grover et al. found that a larger proportion of participants (37.1% and 39.4%, respectively) were hesitant to report incidents of violence due to perceived stress, time-consuming reporting processes, and a lack of knowledge on how to report the events [10, 12]. Singh et al. reported that 50% of doctors believed reporting to be useless [16]. Conversely, Kumar et al. observed a lower proportion of participants who were hesitant to report WPV incidents, although the reasons for not reporting were similar [21].

WPV against doctors has an immense psycho-social impact. Our study showed that WPV negatively affected the mental well-being of 73.3% of participants. In comparison, 69.9% of doctors reported a negative impact on their well-being. It also impacted social life (59.2%) and family (53.4%). These findings were statistically significant (p<0.01), indicating a strong association between WPV and its impact on doctors' lives. Moreover, those who experienced WPV were more likely to consider changing their occupation (p<0.01) and reported decreased motivation (p<0.01). Numerous studies conducted in India indicate that incidents of WPV leave doctors feeling angry, irritable, and frustrated, with some individuals even experiencing depression [14,16]. A growing body of evidence supports the psychological impact of WPV, highlighting the need for interventions that address the mental health consequences of these events [10]. A study by Kumar et al. found that 60% of doctors expressed a desire to change their workplace and work patterns following an episode of violence, while other studies have reported decreased productivity, low morale, and diminished job satisfaction [22-24]. Though India has attained the desired doctor-patient ratio, with the increasing prevalence of non-communicable diseases, the skewed distribution of patients in government hospitals, and the COVID pandemic workload burden, the burden remains high for medical professionals [25,26]. This issue is compounded by the presence of a depressed and anxious workforce, thereby worsening the delivery of healthcare services. Numerous studies carried out in India have documented that physicians frequently experience stress and mental illnesses, which, in some cases, lead to suicide [27,28].

Our study reveals that workplace violence (WPV) against doctors has a significant impact on patient management and clinical decision-making. As a result of violence against doctors, there has been a reduction in the provision of medical and surgical interventions, particularly in emergency situations. However, there has been an increase in the frequency of investigations, consultations with other specialists, referrals, consultation time, and preference for private hospitals over government hospitals. The findings of the study by Kaur et al. were consistent with this study's results, indicating that there was an inverse correlation between the severity of violence experienced by treating physicians and their provision of surgical and medical interventions, as well as their handling of emergency cases. Conversely, prescribing investigations and referrals, as well as consulting with other physicians, were found to increase with WPV [10].

WPV against doctors can have significant psychosocial impacts on the affected healthcare professionals. In addition, it can also result in adverse effects on patient management, such as a reduction in the handling of complex surgical and medical cases. Consequently, this may lead to an increase in referrals and costly investigations, as well as delays in receiving necessary treatment, potentially leading to a worsening of patients' conditions. Furthermore, such delays and suboptimal care can increase dissatisfaction among patients and their relatives, potentially contributing to further incidents of WPV. This vicious cycle needs rather careful consideration.

To prevent workplace violence against healthcare professionals, it is necessary to have strict policies and procedures in place, including effective reporting and surveillance systems. The medical curriculum should also integrate communication skills, and the media should be sensitised to foster mutual understanding. Laws must be enforced to hold perpetrators accountable and rebuild trust in the judicial system, which is crucial for maintaining the doctor-patient relationship and promoting public health.

The present study has certain limitations that should be taken into consideration when interpreting the findings. Firstly, the study design was cross-sectional, and participants were asked to recall experiences of WPV, which may be prone to recall bias. Secondly, the study was conducted in a single tertiary care government hospital, and the results may not be generalizable to other healthcare settings. Despite these limitations, the study used a validated questionnaire specifically designed for the Indian context to assess the prevalence of WPV and its impact on subsequent patient management. The study achieved a 98.7% response rate, indicating a high level of participant engagement.

## Conclusions

The presence of violence in any form is a major concern for society. In the current study, a large proportion of doctors were victims of violence, with verbal aggression being the most frequently reported type. Despite the high incidence of WPV, reporting of these events remains low due to inadequate support and deficient reporting procedures within the healthcare organization. It is clear that violence in healthcare settings has far-reaching consequences that extend beyond the medical professionals themselves, ultimately impacting the quality of healthcare services delivered to the general public. It is imperative that we prioritise the safety and well-being of medical professionals and take steps to prevent violence in healthcare settings.
